# Rationale and design of the Lead Evaluation for Defibrillation and Reliability study: Safety and efficacy of a novel ICD lead design

**DOI:** 10.1111/jce.15747

**Published:** 2023-01-08

**Authors:** George H. Crossley, Prashanthan Sanders, Paolo De Filippo, Khaldoun G. Tarakji, Bert Hansky, Maully Shah, Pamela Mason, Baerbel Maus, Keith Holloman

**Affiliations:** ^1^ Vanderbilt University Nashville Tennessee USA; ^2^ Royal Adelaide Hospital Adelaide South Australia Australia; ^3^ Azienda Ospedaliera Papa Giovanni XXIII Bergamo Italy; ^4^ Department of Cardiovascular Medicine, Cleveland Clinic Cleveland Ohio USA; ^5^ Städtische Kliniken Bielefeld Germany; ^6^ The Children's Hospital Philadelphia Pennsylvania USA; ^7^ University of Virginia Medical Center Charlottesville Virginia USA; ^8^ Bakken Research Center, Medtronic Inc. Maastricht The Netherlands; ^9^ Medtronic, Inc. Mounds View Minnesota USA

**Keywords:** adaptive design, defibrillation lead, virtual patient modeling

## Abstract

**Background:**

Implantable cardioverter defibrillators (ICD) are indicated for primary and secondary prevention of sudden cardiac arrest. Despite enhancements in design and technologies, the ICD lead is the most vulnerable component of the ICD system and failure of ICD leads remains a significant clinical problem. A novel, small‐diameter, lumenless, catheter‐delivered, defibrillator lead was developed with the aim to improve long‐term reliability.

**Methods and Results:**

The Lead Evaluation for Defibrillation and Reliability (LEADR) study is a multi‐center, single‐arm, Bayesian, adaptive design, pre‐market interventional pivotal clinical study. Up to 60 study sites from around the world will participate in the study. Patients indicated for a de novo ICD will undergo defibrillation testing at implantation and clinical assessments at baseline, implant, pre‐hospital discharge, 3 months, 6 months, and every 6 months thereafter until official study closure. Patients may be participating for a minimum of 18 months to approximately 3 years. Fracture‐free survival will be evaluated using a Bayesian statistical method that incorporates both virtual patient data (combination of bench testing to failure with in‐vivo use condition data) with clinical patients. The clinical subject sample size will be determined using decision rules for number of subject enrollments and follow‐up time based upon the observed number of fractures at certain time points in the study. The adaptive study design will therefore result in a minimum of 500 and a maximum of 900 patients enrolled.

**Conclusion:**

The LEADR Clinical Study was designed to efficiently provide evidence for short‐ and long‐term safety and efficacy of a novel lead design using Bayesian methods including a novel virtual patient approach.

## Graphical abstract

The design of the LEADR study is presented. This studies a novel ICD lead that is built on the robust design of the existing Medtronic Model 3830 lead. Both have a flexible central conductor which is designed to make the lead more reliable.

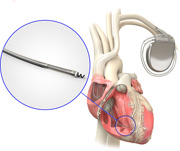

## 1 INTRODUCTION

### 1.1 Rationale

Implantable cardioverter defibrillator (ICD) systems are used for patients who are at risk for sudden cardiac arrest, have exhibited life‐threatening tachyarrhythmia, or have compromised heart function associated with heart failure.^1^ Implantable defibrillators have been available for 40 years with numerous reports on the performance of many defibrillation leads from which to construct performance benchmarks.^2^, ^3^ Despite enhancements in design and technologies, the ICD lead is typically the most vulnerable component of the transvenous ICD system as the annual rate of ICD lead defects that requires intervention increases over time and can reach over 20% at 10 years.^4^, ^5^, ^6^

Lower profile leads with the same or greater reliability can provide alternatives for physicians to serve patients with challenging or small anatomies. Current market‐approved ICD leads have a diameter that varies from 6.8 to 8.6 Fr. (2.27–2.87 mm). Presently available leads use stylet delivery for implantation; necessitating an internal lumen. Previous efforts to decrease ICD lead diameter to allow for ease of implantation and to reduce likelihood of venous obstruction and tricuspid valve regurgitation have resulted in two Food and Drug Administration (FDA)‐mediated recalls. The Medtronic Sprint Fidelis (6.6 Fr) was recalled in 2007 due to its high incidence of conductor fracture and the St. Jude Medical Riata leads (6.3–7.6 Fr) were recalled in 2011 due to insulation failure resulting in externalization of conductor cables.^7^ These recalls caused an assumption that reducing lead diameter might increase lead failure.^8^ However, rather than lead diameter, lead design is believed to be a critical factor for lead performance.^9^, ^10^

The purpose of the LEADR Clinical Study is to demonstrate safety and efficacy of The Model 093000 lead (further referenced as the “LEADR ICD lead”). The performance goals for the primary safety objective and primary efficacy objective (defibrillation testing) are chosen based on the known performance of market‐approved leads or historical study data,^11^ and the objectives are designed to show that the LEADR ICD lead will have acceptable safety and efficacy.

### 1.2 LEAD design

**Figure 1:**
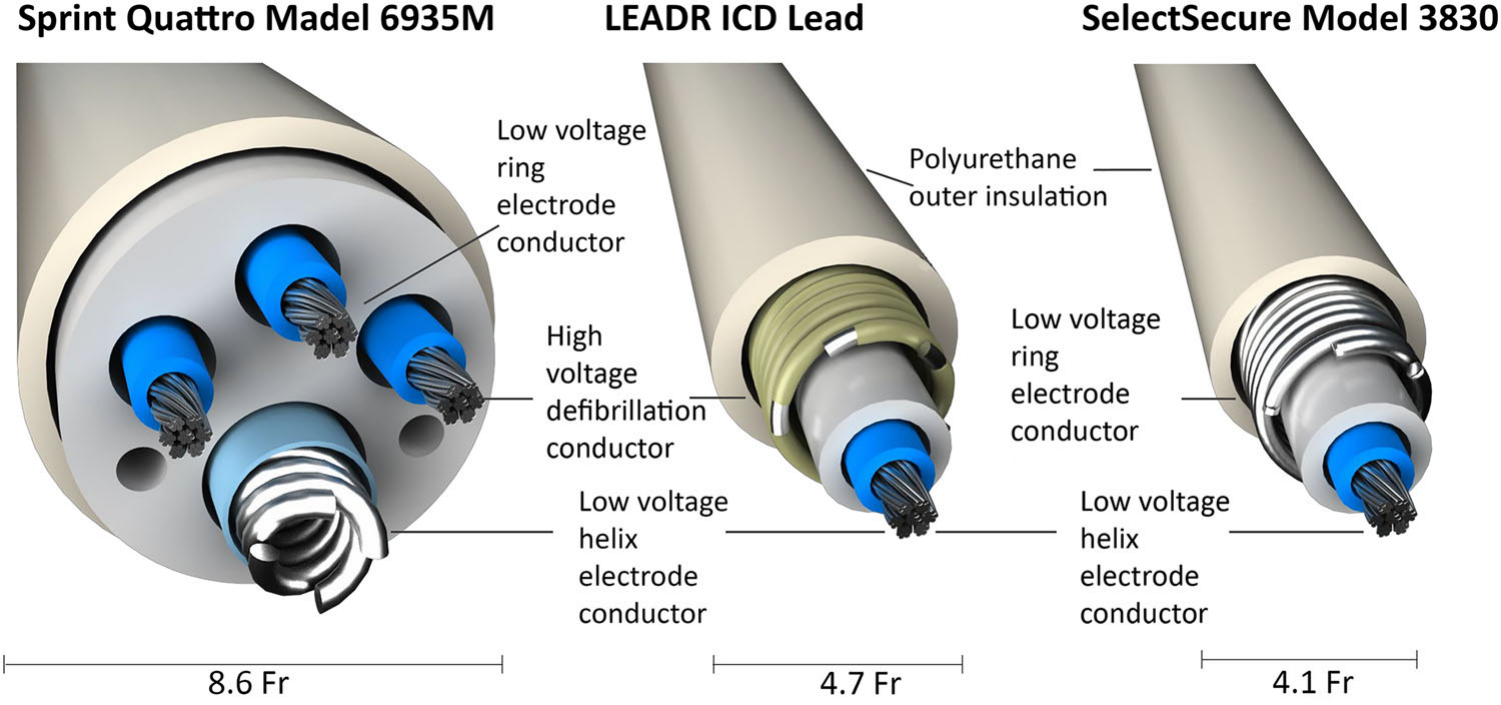
Cross‐sectional comparison of Sprint Quattro Model 6947M, LEADR ICD, and SelectSecure Model 3830 leads. Lead diameter is shown below each lead. Note that the LEADR Lead has thicker outer insulation. Fr, French; ICD, implantable cardioverter defibrillator.

The LEADR ICD lead was developed to address the needs for long‐term reliability and reduced defibrillation lead diameter. This new lead is based on the SelectSecure Model 3830 (Medtronic, Inc.) pacing lead (Figure 1) which received CE mark on January 31st, 2003 and FDA approval on August 3rd, 2005 (ClinicalTrials.govNCT00266682). The SelectSecure Model 3830 (Medtronic, Inc.) pacing lead has long‐term reliability data out to 10 years supporting the design of the LEADR ICD.^12^ Differences of the LEADR ICD lead to the Model 3830 include thicker outer insulation and a polyimide coated, silver cored MP35N outer conductor coil. The polyimide coating provides a second layer of high‐voltage insulation and improved fatigue performance.

(Deleted)The SelectSecure Model 3830 lead is catheter delivered, as opposed to other pacing and defibrillation leads that are delivered using a stylet. The absence of a stylet lumen allows the use of a flexible cable from the connector to the tip, enabling a smaller overall diameter and potential for improved fracture performance due to lower stresses during bending (Figure 1).

The LEADR ICD lead is a 4.7 Fr, steroid‐eluting, catheter delivered, active fixation, polyurethane insulated, integrated bipolar single coil defibrillation lead. The lead is designed for pacing, sensing, cardioversion, and defibrillation therapies. The right ventricular (RV) coil delivers cardioversion and defibrillation therapies. Pacing and sensing occur between the helix and RV coil electrode. The materials in the LEADR ICD lead have been used for more than 10 years in other lead designs with acceptable field performance (Figure S1).^13^

Current ICD lead designs incorporate multiple lumens and conductors to enable both dual and single coil defibrillation therapy as well as integrated and true bipolar sensing. Additionally, current active fixation ICD leads have helices that extend and retract when the conductor coil to the helix is rotated. Although these designs may provide options for physicians, they introduce constraints that can limit durability and increase size.

The LEADR ICD lead architecture is dedicated for single coil defibrillation, integrated bipolar pacing and sensing, and catheter delivery. This eliminates several design constraints that are present in current ICD leads. The single coil defibrillation and integrated bipolar design require only two conductor circuits instead of up to four in traditional designs. Catheter delivery allows the central axis placement of a thin, flexible cable to the tip helix instead of a relatively stiff torque coil that may be more susceptible to fatigue fracture. The cable to the tip helix is placed in the location normally reserved for a stylet lumen, which gives advantages of both low bending stress and size reduction. The conductor to the defibrillation electrode is a coiled helix surrounding the tip conductor, and is also on the central axis, reducing stress compared to coiled helices in traditional ICD leads. Because the outer conductor is coiled around the inner conductor, the cable externalization failure mode seen in other ICD leads is not possible. The LEADR ICD lead design enables improved fatigue strength compared to previous ICD lead designs.

### 1.3 Objectives and study endpoints

**Figure 2:**
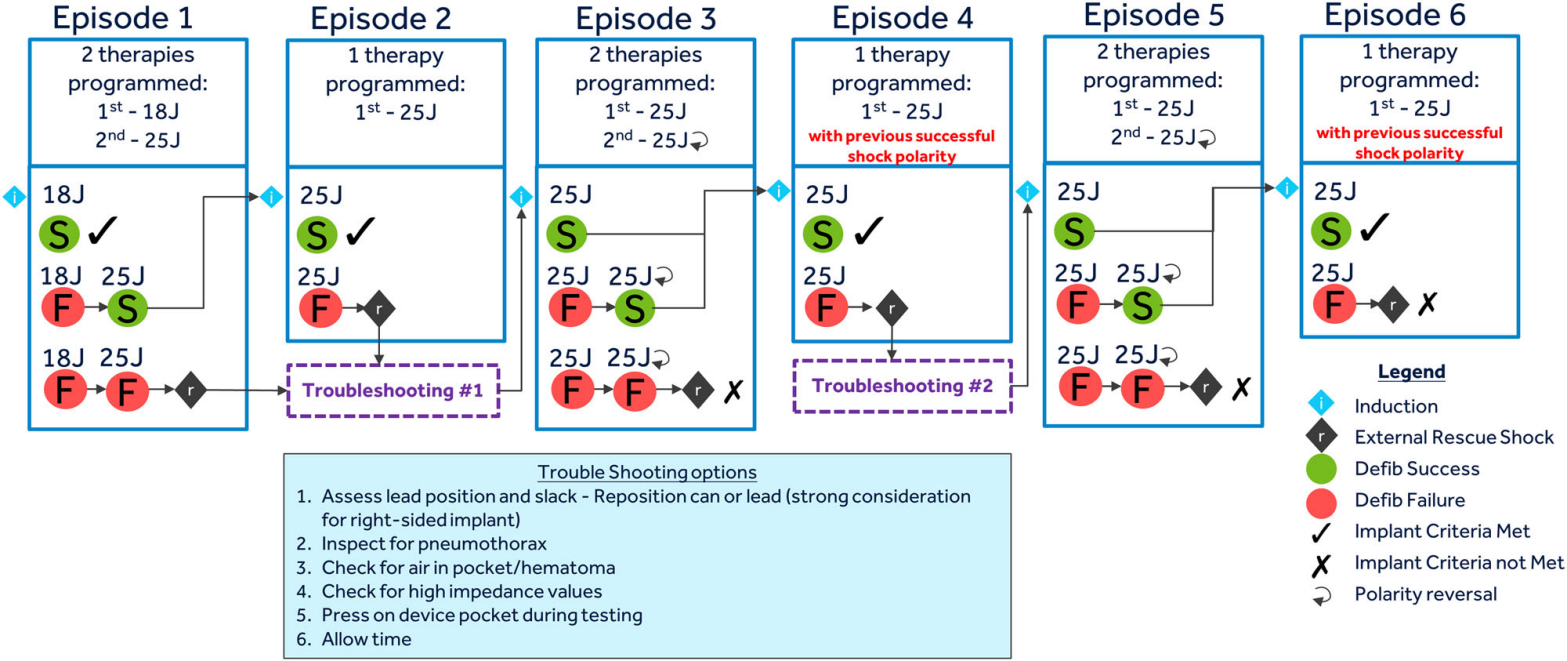
Diagram outlining the process of SSVA induction and defibrillation testing. Depiction of the schematic for defibrillation testing. For defibrillation success, a patient will need to have successful conversion of a single induced sustained shockable ventricular arrhythmia (SSVA) by an 18 J shock or successful conversion of two consecutive induced SSVAs at 25 Joules.

The LEADR study has two primary objectives. The primary safety objective is to demonstrate that the freedom from major complications related to the LEADR ICD lead at 6 months post‐implant exceeds a pre‐specified threshold of 90%. The endpoint is defined as a subject's first occurrence of a major complication related to the LEADR ICD lead, as determined by an independent Clinical Events Committee (CEC), that occurs on or before 6 months (182 days) post‐implant. Major complications are those complications resulting in death, lead fracture, hospitalization, prolongation of an existing hospitalization by at least 48 h, and/or system revision (reposition, replacement, explant). The primary efficacy objective is to demonstrate that defibrillation efficacy exceeds a threshold of 88% in a subset of patients. The defibrillation endpoint will be met if a single 18 Joule shock terminates an induced sustained shockable ventricular arrhythmia (SSVA) or if two consecutive induced SSVAs are terminated with 25 J shocks (Figure 2).

The secondary objective is to estimate the lead fracture‐free rate throughout the study duration. Ancillary objectives are to estimate pacing capture threshold, R‐wave amplitude, and pacing impedance. Finally, adverse events will be summarized over the study duration.

## 2 METHODS

The LEADR Clinical Study is a pre‐market, prospective, multi‐center, single‐arm, Bayesian, adaptive, interventional pivotal clinical study approved by local regulatory and ethics committees that adheres to the Declaration of Helsinki to evaluate efficacy and safety of the LEADR ICD lead (ClinicalTrials.govNCT04863664). The study is expected to be conducted at up to approximately 60 study sites worldwide (Australia, Canada, China, Europe, Hong Kong, Japan, Malaysia, Puerto Rico, Singapore, and the United States). At the time of manuscript submission, 19 sites have been activated and 70 patients have been enrolled.

### 2.1 Study population

At least 500 patients meeting all of the inclusion and none of the exclusion criteria will be enrolled in the study (Table 1). Up to 900 patients may be enrolled dependent on the number of observed fractures based on the adaptive study design. Details about the adaptive design and sample size calculation are presented further below. Patients need to have an indication for implantation of a single or dual chamber ICD or a CRT‐D according to current ACC/AHA/HRS or ESC guidelines.^1^, ^2^, ^3^, ^14^

**Table 1:** Inclusion/exclusion criteria

Inclusion criteria
1. Subject meets current clinical practice guidelines for implantation of ICD or CRT‐D and will undergo one of the following:
*de novo* Medtronic CRT‐D system implant
*de novo* Medtronic ICD system implant (single or dual chamber)
2. Patient has, per local law and requirements, the minimum age for autonomously signing an ICF
3. Patient is willing to undergo implant defibrillation testing if requested.
4. Patient is geographically stable and willing and able to complete the study procedures and visits for the duration of the follow‐up.
Exclusion criteria
1. Patient is unwilling or unable to personally provide informed consent.
2. Patient has contraindications for standard RV transvenous lead placement (e.g., mechanical right heart valve).
3. Patient is contraindicated for ≤1 mg dexamethasone acetate.
4. Patient has a life expectancy of less than 12 months
5. Subject is enrolled or planning to enroll in a concurrent clinical study that may confound the results of this study, (unless pre‐approved).
6. Subject with any exclusion criteria as required by local law (e.g., age or other).
7. Pregnant women or breastfeeding women, or women of childbearing potential and who are not on a reliable form of birth regulation method or abstinence
8. Subject with an existing pacemaker (including transcatheter pacing system), ICD, or CRT device or leads
9. Subject with any evidence of active infection or undergoing treatment for an infection
10. Recent (or planned) cardiac surgery or stenting less than 1 month before implant
11. End‐stage renal disease
12. Patients with NYHA IV classification
13. Patients with a transplanted heart
14. Patients with previously extracted leads
15. Patients with LV Assist Device
Defibrillation testing exclusion criteria:
Pre‐existing or suspected pneumothorax during implant^a^
Current intracardiac left atrial or LV thrombus
Severe aortic stenosis
Severe proximal three‐vessel or left main coronary artery disease without revascularization
Unstable angina
Ejection Fraction less than 25%
Recent stroke or transient ischemic attack (within the last 6 months)
Known inadequate external defibrillation
Any other known medical condition not listed that precludes their participation in the opinion of the investigator

Abbreviations: CRT‐D, cardiac resynchronization therapy‐defibrillator; ICD, implantable cardioverter defibrillator; ICF, informed consent form; LV, left ventricular; NYHA, New York Heart Association; RV, right ventricular.

^a^ Patients meeting this criterion will remain in the study if a LEADR ICD lead has been placed but would be excluded from defibrillation testing.

The first enrollment occurred June 21, 2021. The enrollment period is expected to take approximately 15 months. Individual patients may be participating in the study for a period of minimum 18 months to approximately 3 years. The duration of individual subject participation will vary based on study attrition, timing of study site activation and their enrollment, and the total number of patients to be enrolled which is dependent on the number of fractures observed during the study as described below. Informed consent will be obtained from all subjects.

### 2.2 Data collection

**Figure 3:**
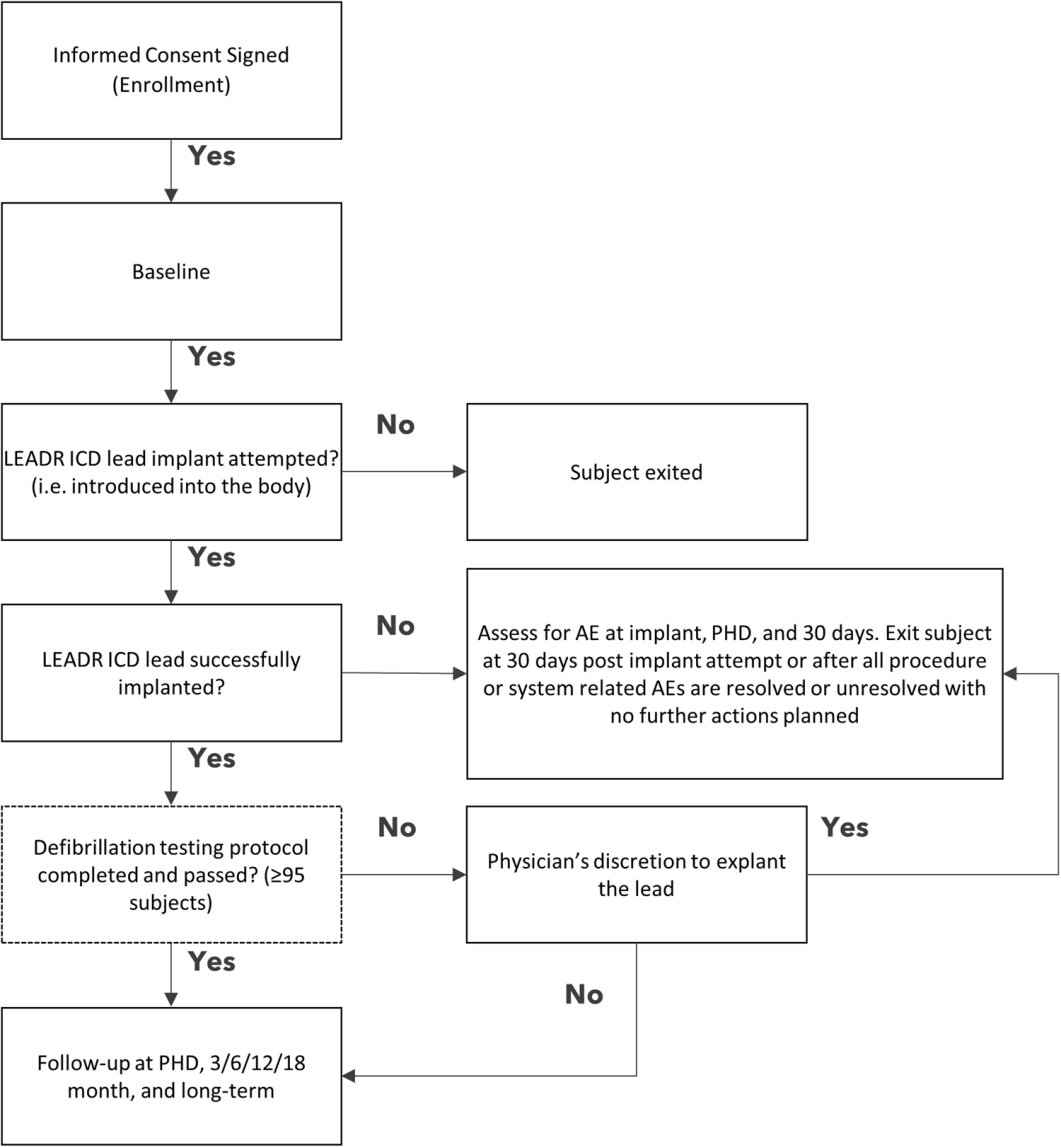
Overview of the LEADR Clinical Study. Study flow diagram. AE, adverse event; ICD, implantable cardioverter defibrillator; PHD, pre‐hospital discharge.

Patients will be implanted with the LEADR ICD lead connected to a Medtronic commercially released MR conditional ICD or CRT‐D device. Any commercially released RA and LV lead may also be implanted. If a lead cannot be successfully implanted, the clinical plan will be determined by the physician. Clinical data will be collected at the study visits detailed in Table 2. At implant, chest X‐ray, fluoroscopic cine images, and analyzer/programmer printouts will be obtained. Defibrillation testing will be performed at the time of implant in enrolled patients until the sample size requirement of at least 95 completed defibrillation testing protocols has been reached. Electrical testing will include R‐wave amplitude and pacing capture threshold testing performed with decrement pacing voltage amplitude starting at 5.0 volts at a fixed pulse width of 0.4 ms. Additionally, lead impedance will be measured. All procedure or system‐related adverse events will be collected, adjudicated, and followed until resolution. For analysis, procedure‐related major complications will be reported separately from other major complications and all complications can be reported in ICD and CRT‐D patient populations separately. Subject visits will occur at enrollment, baseline, implant, pre‐hospital discharge, 3, 6, 12, 18 months post‐implant, and every 6 months thereafter until study closure (Figure 3).

**Table 2:** Data collection and study procedure requirements at subject visits

Study procedure	Enrollment/baseline	Implant	PHD	3/6/12/18 Months (in office)	Long‐term (post‐18 months, remote or in office)	Unscheduled	System modification	Study exit
Informed consent	X							
Inclusion/exclusion assessment	X							
Physical exam, demographics, cardiovascular medical history, surgical history	X							
Physician lead and catheter handling assessment		X					X^a^	
System and procedure information		X					X	
Sensing, Impedance, and pacing tests		X	X	X	X	X^b^	X	
Defibrillation testing		X^c^						
Fluoroscopy of final lead position (LAO)		X					X^a^	
Chest radiographs (AP/PA and lateral)			X				X^a^	
Save‐to‐media/save session files		X	X	X	X	X	X	X
Medications		X						
AEs (including AEs with fatal outcome), device deficiencies, study deviations, lead and systems alerts, lead systems events	As they occur							

*Note*: In‐person visits could be replaced by other options as per required per local regulations in the case of, e.g., a pandemic.

Abbreviations: AE, adverse events; AP, anteroposterior; LAO, left anterior oblique; PA, posteroanterior; PHD, pre‐hospital discharge.

^a^ Only required in case of replacement of the LEADR ICD lead.

^b^ Optional.

^c^ Required for a minimum of 95 patients.

### 2.3 Induction and defibrillation testing

To estimate the LEADR ICD lead defibrillation testing success rate (primary efficacy objective), sustained shockable ventricular arrhythmia (SSVA) induction and defibrillation testing is required at implant for at least the first 95 consecutive implanted patients. Only a subset of patients is required to undergo defibrillation testing so as to reduce risk and burden to the patients (Figure 2). Additional exclusion criteria (Table 1) will apply to patients for defibrillation testing, to reduce the risk of complications when contraindications are documented.^15^

### 2.4 Adaptive design and sample size

**Figure 4:**
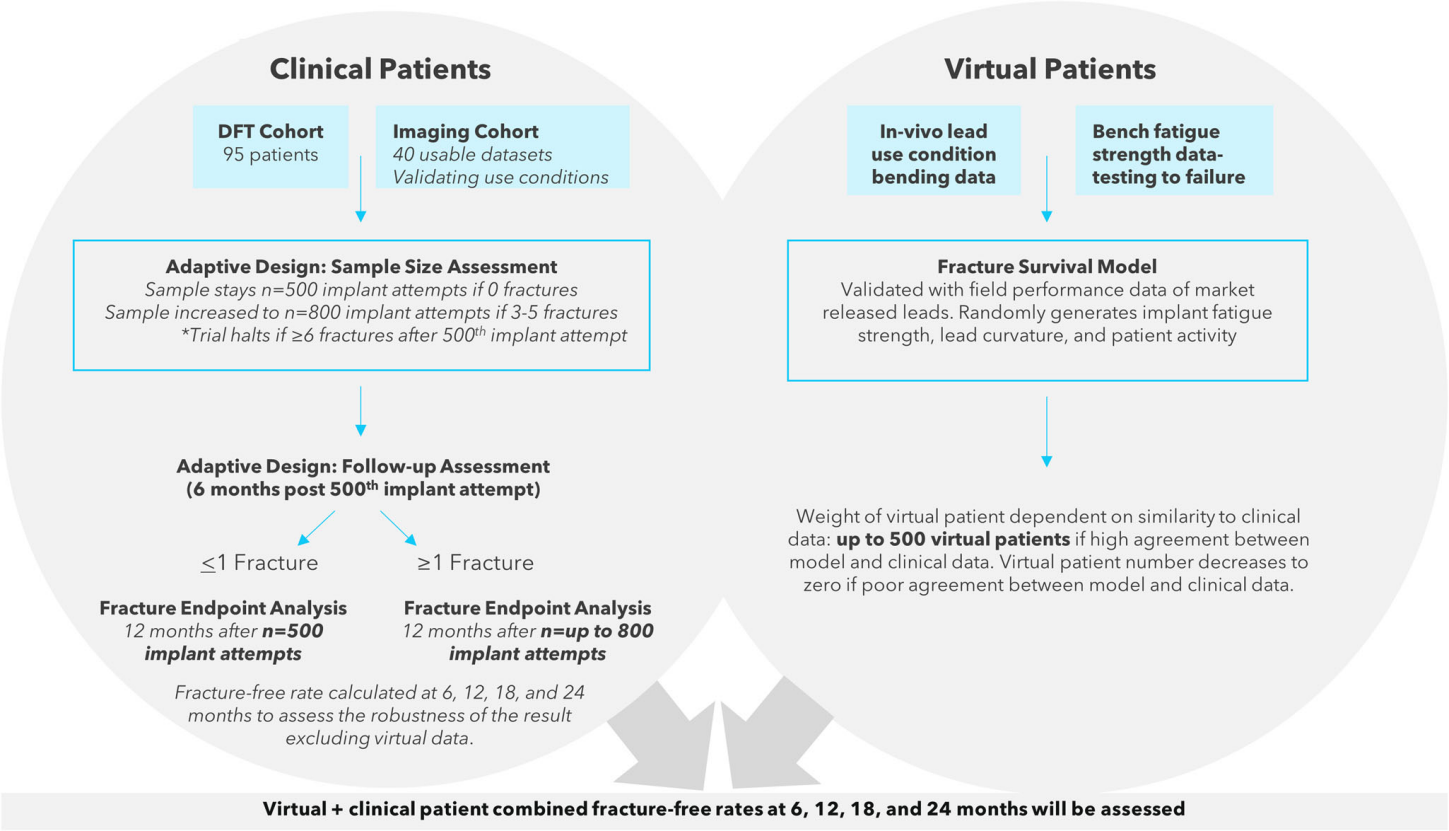
Bayesian and adaptive design combines virtual + clinical patients for analysis of conductor fractures. The Bayesian and adaptive study design is shown for both the clinical and virtual patient cohorts. DFT, defibrillation testing.

The LEADR Clinical Study design is adaptive with regard to both sample size and follow‐up duration (Figure 4). It features two decision points based on LEADR ICD lead fractures observed in the study, which are an important component of the primary safety endpoint and a key measure of lead reliability. At these decision points, only the number of fractures will be assessed. Based on the decision rules (Table 3) the sample size may increase from 500 to up to 900 patients.

**Table 3:** Adaptive design decision rules and operating characteristics

Stage	Time point	Decision rule	Chance of occurrence	
			LEADR ICD lead	Fidelis‐like Lead
Sample size assessment	500th implant attempt	0 fractures observed: halt enrollment	85.7%	10.1%
		1 fracture observed: increase to 600 implant attempts	13.4%	23.4%
		2 fractures observed: increase to 700 implant attempts	0.8%	26.5%
		3–5 fractures observed: increase to 800 implant attempts	0.03%	37.1%
		6+ fractures observed: halt study	0.0%	2.9%
Follow‐up Assessment	6 months after 500th implant attempt	≤1 fracture observed: analyze at 12 months post‐500th implant attempt	94.3%	1.4%
		>1 fracture observed: analyze at 12 months post‐last implant attempt	5.7%	98.6%

Abbreviation: ICD, implantable cardioverter defibrillator.

The first decision point occurs after 500 subject implant attempts (Table 3). If no fractures (defined as a fracture of one or more conductor filars and adjudicated by an independent CEC) have been observed at that time point, enrollment will be halted, and all enrolled patients will be followed. If instead six or more fractures have been observed, the study will be halted for safety. If one to five fractures have been observed, the sample size will be increased accordingly (Table 3). To further account for patients who enroll in the study but exit before an implant attempt, up to 900 patients may be enrolled.

The second decision point will be when the first 500 patients with an implant attempt have had the opportunity to have a 6‐month visit. If at that time point, no more than one fracture has been observed in the study, the data will be analyzed when the first 500 patients with an implant attempt have had the opportunity to be followed for 12 months. If instead more than one fracture has been observed by this time point, the data will be analyzed once the last (e.g., 600th, 700th, 800th) implanted subject has had the opportunity to reach a 12‐month follow‐up.

For analyzing the primary safety objective, a piecewise exponential survival model employing Gamma priors which yield a skeptical prior distribution for a 6‐month freedom rate (median of 56.2%) is used. The posterior distribution will be computed using the conjugate distribution for the Gamma priors. Information about simulations to study the statistical operating characteristics of the adaptive design and details about sample size calculations for the primary efficacy objective can be found in the supplementary information.

Several simulation studies using the statistical programming language R v4.0.2 (r-project.org) were performed to study the statistical operating characteristics of the adaptive design. For all simulations, an attrition rate of 4% at 1 month and 10% 1‐year post‐implant were assumed. Power for the primary safety objective was estimated by assuming a major complication‐free rate of 95.5% and 94.5% at 1 and 6 months, respectively. Simulation results showed that the adaptive study design has good frequentist operating characteristics, such as power >90% and Type I error <2.5% for the primary safety objective (Table S1).

Additionally, simulations were carried out under two different scenarios for the secondary objective to assess ensure the adaptive design could differentiate between a high‐performing and low‐performing lead. Specifically (1) a high‐performing scenario where the LEADR ICD lead has an expected fracture‐free rate of 99.9% at 12 months, and (2) a poor‐performing scenario where the lead has a 98.4% fracture‐free rate at 12 months. In the high‐performance scenario, there is a 94% chance of observing 1 or fewer fractures within 6 months after the 500th implant attempt, compared to 1.4% chance for the poor‐performing scenario.

The primary efficacy objective will be analyzed using a Beta‐Binomial Bayesian model with a skeptical Beta before ensure good Frequentist operating characteristics. To determine the sample size for the primary efficacy objective, a defibrillation success rate of 97% was assumed to study statistical power. To assess the Type I error, a success rate of 88% was assumed. Simulation studies determined that at least 95 patients completing the defibrillation testing protocol will provide a power >90% with a Type I error <2.5%.

### 2.5 Preclinical testing and virtual patient simulation

The secondary objective of fracture‐free survival will be evaluated using a novel Bayesian statistical method that incorporates modeled and clinical data (Figure 4).^16^ To generate modeled data, individual patients are simulated by randomly generating an implant fatigue strength, an implanted lead curvature, and a patient activity level. The expected time to fracture is calculated from these values. The process is repeated many times to simulate a cohort of so‐called virtual patients that can be used to construct an informative prior distribution for fracture survival.^17^ The model was validated using field data for market‐released Quattro and Fidelis leads. The LEADR ICD lead has shown very high fatigue strength in established methods^15^, ^18^, ^19^, ^20^, ^21^ that are designed to mimic the cyclic bending experienced in the shoulder and the heart in bench tests. Fatigue strength data along with in‐vivo lead bending data (imaging clinical data cohort) will be combined^19^ to model fracture survival in the LEADR study. As a result of fatigue testing and imaging of patients with the SelectSecure Model 3830 lead, the current expected fracture survival of the LEADR ICD lead is 99.8% at 24 months. This result will be updated with in‐vivo lead bending data measured from biplane fluoroscopic images from a subset of patients in the LEADR study (imaging clinical data cohort). The modeled fracture survival will be combined with clinical data collected during the LEADR study according to pre‐specified decision rules. Briefly, the virtual patient sample size is limited to 500 patients, and the impact of these virtual patients adjusted based upon the agreement with the clinical data. If the data agree, the impact of the virtual patients will be greater than if there is less agreement. Furthermore, the fracture endpoint will be evaluated with and without the use of virtual patients.

### 2.6 Analysis methods

Bayesian analysis methods will be used for all objectives.

For the primary safety objective, the null hypothesis is that the 6‐month freedom from major RV lead‐related complications is equal to or below 90%. For the safety objective to be successfully met, the 95% two‐sided credible interval for the 6‐month RV lead‐related major complication rate must have a lower bound exceeding 90%.

The primary safety objective addresses the entire safety profile of the LEADR ICD lead as fractures are included in major complications. The determination of the pre‐specified performance goal of 90% for the lead‐related major complication endpoint aligns with previous ICD and CRT‐D studies: Block HF, Reverse, Evera MRI, and PainFREE SST.^22^, ^23^, ^24^, ^25^ An assessment of comparable major complication‐free rates at 6 months for transvenous devices showed observed rates as low as 95.7% (Figure S2).

For the primary safety objective, all patients undergoing an implant attempt of the LEADR ICD lead will be included in the analysis. The objective will be analyzed using a time‐to‐event analysis. The time to event will be defined as a subject's time to first LEADR ICD lead‐related major complication. In the case of no event, patients will be censored at the time point of their last exposure to the LEADR ICD lead.

For inferential analyses, a piecewise exponential model will be assumed with two intervals of interest: 0‐1‐month post‐implant, and 1 month and beyond post‐implant, as it is possible the rate of lead‐related major complications may differ in the acute (first 30 days) compared to more chronic time points (Figure S2).

Bayesian Prior Gamma distributions will be combined with the prospective event/follow‐up data to generate posterior distributions for each event rate. These posterior distributions will be used to construct the 95% two‐sided credible interval for the freedom from major complication at 6‐months as described above. Further details about the specification of the Bayesian priors can be found in the supplementary methods.

For the primary efficacy objective, the null hypothesis is that the implant defibrillation success rate is equal to or below 88%. For the objective to be successfully met, the 95% two‐sided credible interval must have a lower bound that exceeds 88%. The analysis cohort will be defined as patients who complete the implant defibrillation testing protocol.

Defibrillation implant success for transvenous devices, though no longer routinely performed, has historically been shown to be as low as 88%.^26^ The threshold of 88% has also been used in pivotal trials for alternative defibrillation devices (subcutaneous ICD^27^ and extravascular ICD^28^), demonstrating a precedent for a performance goal of 88%.

The primary efficacy objective will be analyzed using a Beta‐Binomial Bayesian model as described above in the sample size section from which a 95% two‐sided credible interval for defibrillation success will be constructed. Details can be found in the supplementary methods.

For the secondary objective analyses, time‐to‐event analyses will be performed. Time to event will be set as the interval from the time of implant to the time of the first LEADR ICD lead fracture. Patients without fractures during the time period of interest will be censored at their time point of last exposure to the LEADR ICD lead. All patients undergoing an implant attempt of the LEADR ICD lead will be included in the analysis. More information about the analysis for the secondary objective can be found in the supplementary information.

For the secondary objective analyses, time‐to‐event analyses will be performed. Time to event will be set as the interval from the time of implant to the time of first LEADR ICD lead fracture. Patients without fractures during the time period of interest will be censored at their time point of last exposure to the LEADR ICD lead. All patients undergoing an implant attempt of the LEADR ICD lead will be included in the analysis. More information about the analysis for the secondary objective can be found in the supplementary information.

### 2.7 Study management and event adjudication

The sponsor will manage data collection and monitoring. An independent CEC will be utilized to regularly review and adjudicate reported system, procedure‐related adverse events, and deaths. An Episode Review Committee containing independent reviewers will be utilized to review and adjudicate device‐treated episodes to determine appropriateness of therapy (shocks and anti‐tachycardia pacing) delivered to patients. Overall oversight will be provided by an independent Data Monitoring Committee to review accumulating data, help safeguard the interests of study patients and monitor the overall conduct of the study.

## 3 DISCUSSION

Prior efforts to design a small‐diameter defibrillation lead have been met with unexpected challenges that have led to failure based upon the mechanical properties of the leads.^29^, ^30^ Since then, physicians and industry engineers have recognized the interaction between lead design, implant technique, and patient factors, resulting in new methods for evaluating lead fracture. These methods incorporate both bench test results and in‐vivo flexural use conditions to predict performance.^17^, ^21^, ^23^ The design of the LEADR ICD lead is based upon a pacemaker lead (Medtronic SelectSecure Model 3830) that was commercially released in 2003 and has demonstrated 97% survival at 110 months for RV implant locations, showing that a small diameter, lumenless lead can be designed for reliability.^13^ The LEADR study has a Bayesian design that will leverage virtual patient data from an engineering model for fracture survival and use a discount function to adjust the influence of the model based on level of agreement with the clinical data and virtual patient data. There are multiple advantages with the use of such a Bayesian approach. The inclusion of virtual patient data will allow for increased precision of the credible intervals for the fracture‐free survival rate assuming that both data sources agree, while reducing the number of clinical patients to be enrolled to obtain such precision. Before the start of a clinical study, the fracture survival engineering model was used to characterize expected lead performance before human implant, showing performance comparable to existing Medtronic ICD leads. This Bayesian approach allows to efficiently make use of prior information which has been developed over several years by extensive testing, modeling, and analysis of post‐market surveillance data and product returns. If the clinical and virtual patient data agree based on the data collected during the follow‐up time frame of the study, the fracture survival engineering model can be extended to several years of follow‐up and also provide indication about the expected fracture survival rate beyond the follow‐up duration of the study. Additionally, the adaptive nature of the design will allow stopping early, if necessary, for safety reasons. The long‐term performance of the LEADR ICD lead, including fractures, will also be assessed through a post‐market surveillance study.

In addition to assessing the safety, and fracture survival of the LEADR ICD lead, defibrillation efficacy will also be analyzed. As defibrillation testing is no longer routine at many centers,^31^ this study employs a unique testing scheme depicted in Figure 2 that limits the number of shocks needed by defining success as either a single SSVA conversion at 18 J or two consecutive SSVA conversions at 25 J. This testing scheme allows for confirmation of defibrillation efficacy while limiting patient exposure to unnecessary shocks.

## 4 CONCLUSION

The LEADR Clinical Study was designed to efficiently provide evidence for short and long‐term safety and efficacy using Bayesian methods including a novel virtual patient approach. A positive study, meeting our endpoints, would demonstrate the clinical safety and efficacy of this technology to provide physicians an option for a smaller diameter lead in treating patients indicated for an ICD or CRT‐D.

## FUNDING STATEMENT

This study is funded by Medtronic, Inc.

## Supporting information

Supplementary information.

## Data Availability

The data that support the findings of this study are available on request from the corresponding author. The data are not publicly available due to privacy or ethical restrictions.
