# Supplementary material for: Rationale and design of the Lead Evaluation for Defibrillation and Reliability study: Safety and efficacy of a novel ICD lead design
Source: J Cardiovasc Electrophysiol. 2023 Jan 8;34(2):257–67. doi: 10.1111/jce.15747 (PMC10107290; doi:10.1111/jce.15747)
Supplement: Supplementary file 1 — Supplementary information. [file JCE-34-257-s001.docx]

**Supplement to: Rationale and Design of the Lead EvaluAtion for Defibrillation and Reliability (LEADR) Trial: Safety and Efficacy of a novel ICD lead design**

George H Crossley, MD, FHRS, FACC^a^; Prashanthan Sanders, MBBS, PhD^b^; Paolo De Filippo, MD^c^; Khaldoun G. Tarakji, MD, MPH^d^; Bert Hansky, MD^e^; Maully Shah MD^f^; Pamela Mason, MD^g^; Baerbel Maus, PhD^h^; Keith Holloman, BS^i^

Affiliations: ^a^Vanderbilt University, Nashville, TN, USA; ^b^Royale Adelaide Hospital, Adelaide, Australia; ^c^Azienda Ospedaliera Papa Giovanni XXIII , Bergamo, Italy; ^d^Department of Cardiovascular Medicine, ^e^Cleveland Clinic, Cleveland, Ohio , USA; ^e^Städtische Kliniken Bielefeld , Germany; ^f^The Children’s Hospital, Philadelphia, PA, USA; ^g^University of Virginia Medical Center , Charlottesville, VA, USA; ^h^Bakken Research Center, Medtronic Inc, Maastricht, The Netherlands; ^i^Medtronic, Inc, Mounds View, Minnesota

# **Adaptive Design & Sample Size**

Several simulation studies using the statistical programming language R v4.0.2 (r-project.org) were performed to study the statistical operating characteristics of the adaptive design. For all simulations, an attrition rate of 4% at one month and 10% one-year post-implant were assumed. Power for the primary safety objective was estimated by assuming a major complication free rate of 95.5% and 94.5% at 1-month and 6-months respectively. Simulation results showed that the adaptive study design has good frequentist operating characteristics, such as power > 90% and type I error < 2.5% for the primary safety objective (Supplementary Table 1).

Additionally, simulations were carried out under two different scenarios for the secondary objective to ensure the adaptive design could differentiate between a high performing and low performing lead. Specifically, 1) a high performing scenario where the LEADR ICD lead has an expected fracture free rate of 99.9% at 12 months, and 2) a poor performing scenario where the lead has a 98.4% fracture free rate at 12-months. In the high-performance scenario, there is a 94% chance of observing 1 or fewer fractures within 6 months after the 500th implant attempt, compared to 1.4% chance for the poor performing scenario.

**Supplementary Analysis Methods:**

For analysis of the primary safety objective, two informative Gamma priors will be used, one prior for the hazard rate in the first month and one prior for the hazard rate for one-month post-implant and beyond. The priors were chosen to allow for a higher event rate in the first month compared to after the first month as seen in previous studies (see Figure 4), to control frequentist type 1 error and to result in a skeptical prior distribution for the 6-month major complication free rate. Specifically, the prior distributions specify approximately one major complication in 3.5 patient months in the first month, and one major complication in 12 patient months for the remaining time. The posterior distributions will be utilized to determine the posterior distribution for the 6-month freedom from Next Generation ICD lead -related major complication rate. From this distribution a 95% two-sided credible interval will be generated.

A simulation study was performed to determine the power and type I error control of the Bayesian methodology under the alternative and null hypothesis. The simulation study incorporated the adaptive nature of the study allowing the sample size to adapt based on the number of lead fractures observed as described in Table 3.

The simulation study included scenarios under the null hypothesis where the functional form of the piecewise exponential model was mis-specified, e.g., a different cut-off point for the hazard rate than one month was assumed. For each scenario 10,000 simulations of the study were simulated. Subjects were enrolled at a rate of up to 4 subjects per center per month with a most likely value of 1 with a total of 47 centers activating over a period of 13 months.

Supplemental Table 1 shows that under the planned alternative hypothesis where the assumed freedom from RV related major complications is 95.5% at 1-month post-implant and 94.5% at 6-months post-implant the power of the study is 94.4%. Under all null hypothesis scenarios studied, the type I error rate was <2.5% indicating that the type I error rate is well controlled.

For the primary efficacy objective, the prior was chosen to control frequentist type I error and results in a skeptical prior distribution for the defibrillation success rate, i.e., the prior distribution specifies that the average success rate of a patient to pass the defibrillation protocol would be 45%. Combining the collected implant defibrillation data and the prior distribution, a Beta posterior distribution will be generated to determine a posterior mean and 95% two-sided credible interval for the proportion of subjects who successfully pass the defibrillation protocol.

For the secondary objective of fracture analysis, aa piecewise exponential model will be assumed with two intervals of interest: 0-12 months post-implant, and 12 months and beyond post-implant, as it is possible the rate of fractures may vary between the first and second 12-month periods. Non-informative prior Gamma distributions will be used for the fracture hazard rates during these two time periods.

The virtual patient data will be incorporated in the form of a discount prior Gamma distribution for the hazard rate during the first 12 months and a discount prior gamma distribution for the hazard rate after 12 months.16 The influence of each prior on the final study results will be down weighted by two factors for each interval:

ρ=degree of agreement between the Gamma distribution and the posterior for the hazard rates, and

δ= proportion of clinical follow-up data obtained relative to the minimum planned sample size of 500 patients with an implant attempt (δ will not exceed 1 in the case more than 500 years of follow-up are accrued during an interval). Therefore, the maximum influence of virtual patients in the prior distribution is equivalent to 500 clinical patients in the case of high agreement between model and clinical data. The minimum influence of virtual patients is zero, in the case of poor agreement between model and clinical data. In this case the estimated fracture rates would be based solely on the observed clinical data.

The actual parameter values for the virtual patient priors will be determined based on the lead fracture survival engineering model described above.
Posterior distributions for the hazard rates will be determined combining the observed clinical follow-up with the virtual patient priors and non-informative Gamma priors. The final posterior distribution for the hazard rates will be used to generate corresponding posterior distributions and 95% credible intervals for the fracture-free rate at 6, 12, 18 and 24 months.

An additional analysis will be performed without the use of virtual patients to study how the virtual patients influence the results.

**Supplemental Tables**

**Supplemental Table 1. Power and Frequentist Type I Error Control for the Primary Safety Objective**

| **Scenario** | **Assumptions** | **Probability of Rejecting Null Hypothesis** |
| --- | --- | --- |
| Planned alternative hypothesis | Major complication free rate of 95.5% at 1-month post-implant and 94.5% at 6-months post-implant | 0.944 |
| Next Generation lead performs worse than expected, but better than null scenario | Major complication free rate of 95.5% at 1-month post-implant and 92.0% at 6-months post-implant | 0.255 |
| Null hypothesis scenario #1 | Major complication free rate of 95.5% at 1-month post-implant and 90% at 6-months post-implant | 0.017 |
| Null hypothesis scenario #2 | Major complication free rate of 91.0% at 15-days post-implant and 90% at 6-months post-implant | 0.013 |
| Null hypothesis scenario #3 | Major complication free rate of 90.5% at 5-months post-implant and 90% at 6-months post-implant | 0.015 |

**Supplementary Figures**

**Supplementary Figure 1.**


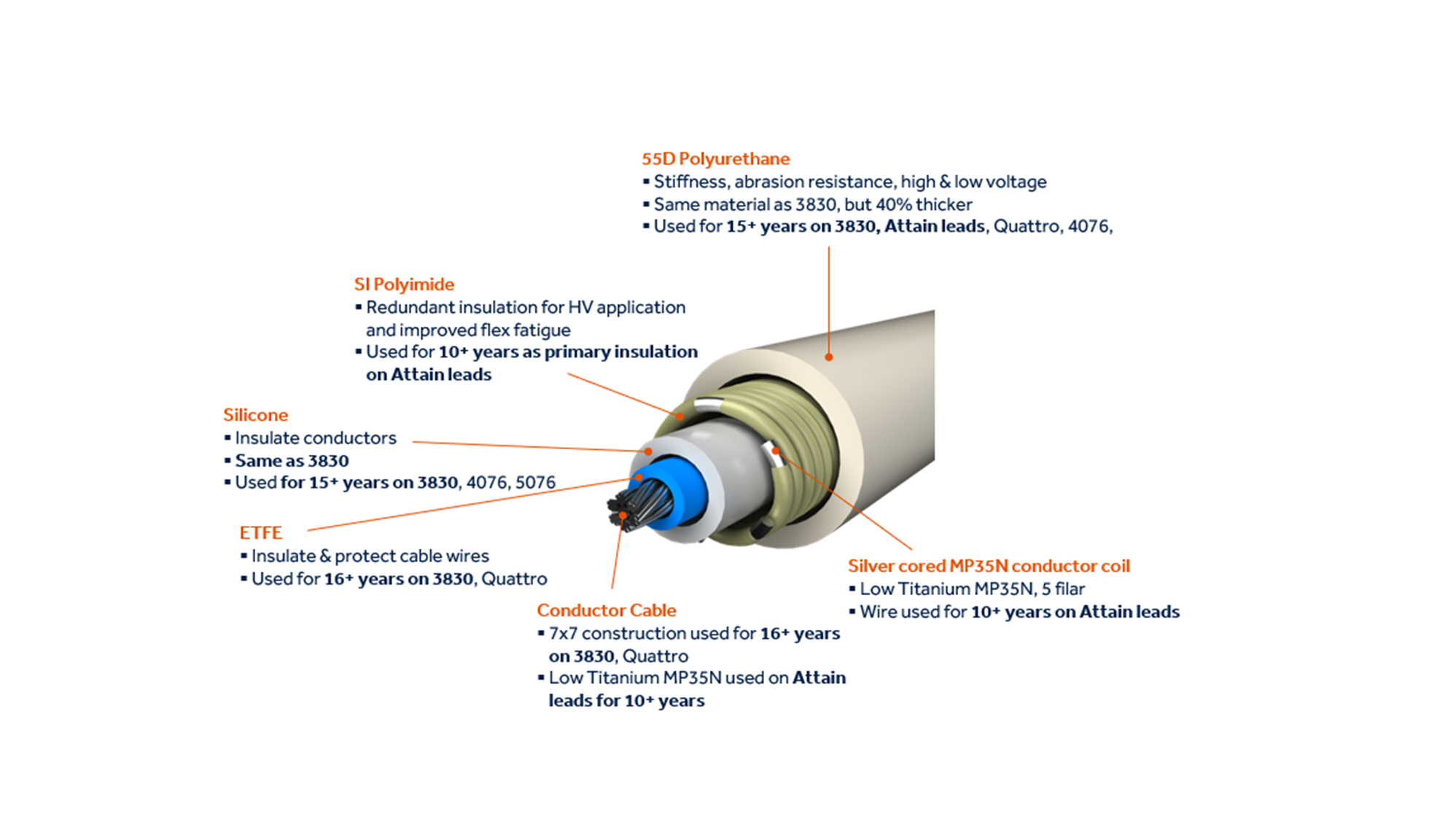


Supplementary Figure 2: RV lead-related Major Complication-Free Rates in Previous Medtronic ICD and CRT-D Studies. The complication-free rate of leads from other Medtronic studies are shown over time. ICD = implantable cardioverter defibrillator; CRT-D = cardiac resynchronization therapy-defibrillator; MRI = magnetic resonance imaging.


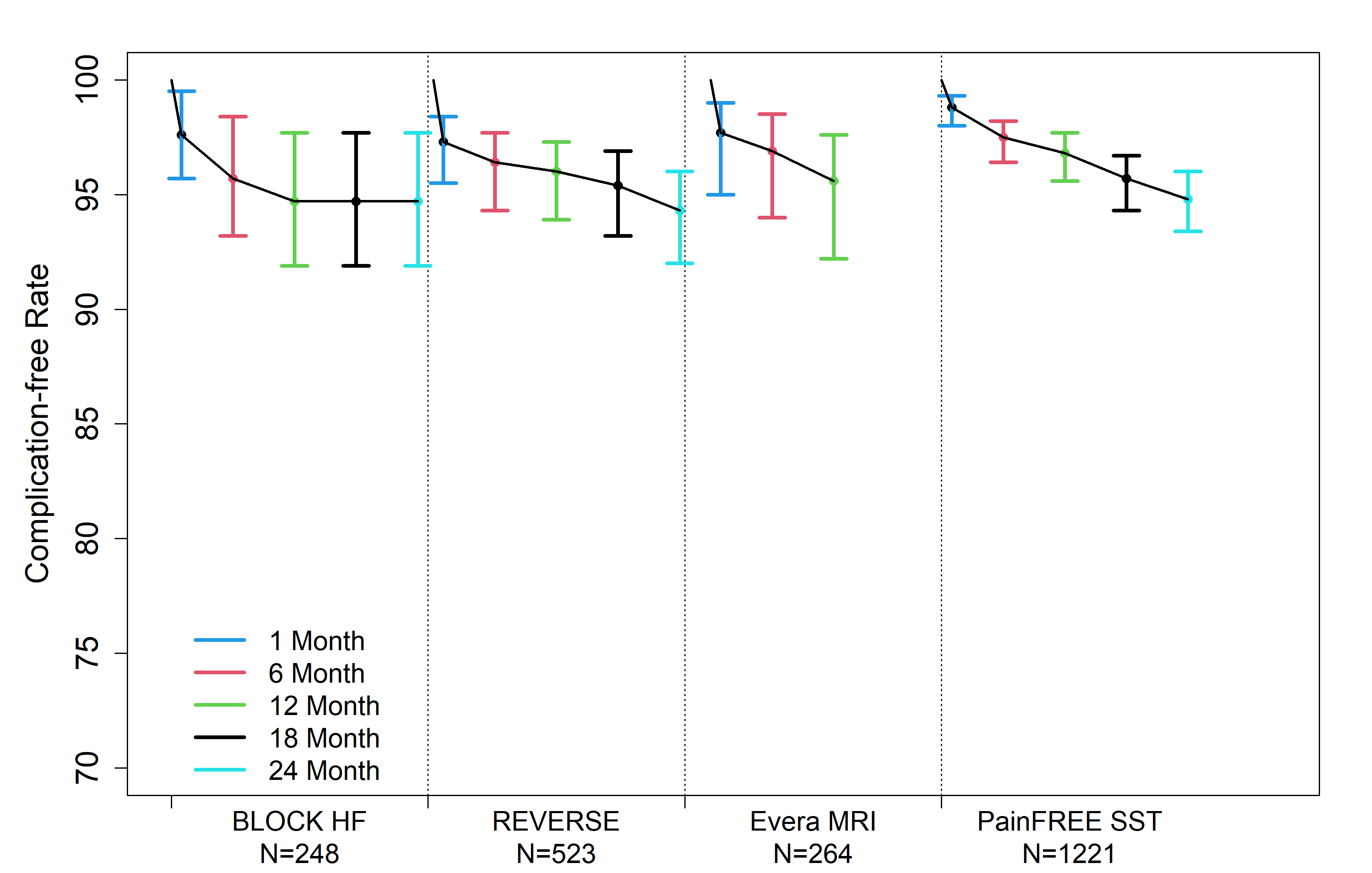


*Analysis cohorts from these studies were restricted to include patients with ICD or CRT-D devices. Patients with successful and unsuccessful implant attempts were included.
